# 

**Published:** 1985-01

**Authors:** 


					CHIRURGICAL
JANUARY 1985
VOLUME 100 (i)
NUMBER 373

				

